# Relationship between Environmental Phthalate Exposure and the Intelligence of School-Age Children

**DOI:** 10.1289/ehp.0901376

**Published:** 2010-03-01

**Authors:** Soo-Churl Cho, Soo-Young Bhang, Yun-Chul Hong, Min-Sup Shin, Boong-Nyun Kim, Jae-Won Kim, Hee-Jung Yoo, In Hee Cho, Hyo-Won Kim

**Affiliations:** 1 Division of Child and Adolescent Psychiatry, Department of Psychiatry and Institute of Human Behavioral Medicine, Seoul National University College of Medicine, Seoul, Republic of Korea; 2 Department of Psychiatry, Ulsan University Hospital, University of Ulsan College of Medicine, Ulsan, Republic of Korea; 3 Department of Preventive Medicine, Seoul National University College of Medicine and Institute of Environmental Medicine, Seoul National University Medical Research Center, Seoul, Republic of Korea; 4 Department of Psychiatry, Seoul National University Bundang Hospital, Seongnam, Republic of Korea; 5 Department of Psychiatry, Gil Medical Center, Gachon University of Medicine and Science, Incheon, Republic of Korea; 6 Department of Neuropsychiatry, Dongguk University International Hospital, Dongguk University College of Medicine, Goyang, Republic of Korea

**Keywords:** children, cognition, dibutyl phthalate (DBP), di(2-ethylhexyl)phthalate (DEHP), IQ, mono-2-ethylhexyl phthalate (MEHP), mono(2-ethyl-5-oxohexyl) phthalate (MEOHP), mono-*n*-butyl phthalate (MBP), phthalate, sex

## Abstract

**Background:**

Concern over phthalates has emerged because of their potential toxicity to humans.

**Objective:**

We investigated the relationship between the urinary concentrations of phthalate metabolites and children’s intellectual functioning.

**Methods:**

This study enrolled 667 children at nine elementary schools in five South Korean cities. A cross-sectional examination of urine phthalate concentrations was performed, and scores on neuropsychological tests were obtained from both the children and their mothers.

**Results:**

We measured mono-2-ethylhexyl phthalate (MEHP) and mono(2-ethyl-5-oxohexyl)phthalate (MEOHP), both metabolites of di(2-ethylhexyl)phthalate (DEHP), and mono-*n*-butyl phthalate (MBP), a metabolite of dibutyl phthalate (DBP), in urine samples. The geometric mean (ln) concentrations of MEHP, MEOHP, and MBP were 21.3 μg/L [geometric SD (GSD) = 2.2 μg/L; range, 0.5–445.4], 18.0 μg/L (GSD = 2.4; range, 0.07–291.1), and 48.9 μg/L (GSD = 2.2; range, 2.1–1645.5), respectively. After adjusting for demographic and developmental covariates, the Full Scale IQ and Verbal IQ scores were negatively associated with DEHP metabolites but not with DBP metabolites. We also found a significant negative relationship between the urine concentrations of the metabolites of DEHP and DBP and children’s vocabulary subscores. After controlling for maternal IQ, a significant inverse relationship between DEHP metabolites and vocabulary subscale score remained. Among boys, we found a negative association between increasing MEHP phthalate concentrations and the sum of DEHP metabolite concentrations and Wechsler Intelligence Scale for Children vocabulary score; however, among girls, we found no significant association between these variables.

**Conclusion:**

Controlling for maternal IQ and other covariates, the results show an inverse relationship between phthalate metabolites and IQ scores; however, given the limitations in cross-sectional epidemiology, prospective studies are needed to fully explore these associations.

Phthalates are mostly used as plasticizers or softeners in polyvinyl chloride (PVC) or vinyl. Environmental chemicals such as phenols, phthalates, and dioxin-like compounds are endocrine disruptors that show estrogen-like activity and may alter normal brain development (Tanida et al. 2009).

Although there are many data on the effects of these chemicals on animals (Shea 2003), there is little convincing evidence of adverse effects in humans (Duty et al. 2005; Hauser et al. 2006; Kamrin 2009); however, there are some reports on the effects of phthalates on children’s health (Sathyanarayana 2008), including the development of the reproductive system (Lottrup et al. 2006), reduced birth weight (Wolff et al. 2008), and positive associations with allergy and asthma (Bornehag et al. 2004; Kolarik et al. 2008).

There is also growing concern about the adverse effects of phthalates on neurodevelopment. In recent years, there has been a growing concern about mental conditions, ranging from cognitive impairment to schizophrenia, that have been linked to endocrine disruption (Brown 2009). Experimental studies suggest that higher levels of di(2-ethylhexyl)phthalate (DEHP) may have adverse effects on neurobehavioral parameters in mice (Tanaka 2002, 2005). Environmental exposure to phthalates in humans may contribute to poor neurodevelopmental outcomes. Few studies have reported an association between prenatal phthalate exposure and neurologic effects in humans. Recently, Engel et al. (2009) reported variable associations between individual phthalate metabolites or their molar sums and many developmental scores; the researchers also found a significant interaction between phthalate metabolites and gender.

An analysis of our preliminary 2007 survey data on school-age children revealed that environmental levels of lead and manganese may have a negative synergistic effect on the intelligence of school-age children (Kim et al. 2009b). We also observed a positive association between phthalate metabolites in urine and symptoms of attention deficit hyperactivity disorder (ADHD) (Kim et al. 2009a), but we were unable to reach a definite conclusion on the association between phthalate and the cognitive functioning of the subjects. This may have been due to the limitations of our 2007 survey; the sample size was small, and we had no information regarding the intelligence quotient (IQ) of the subjects’ mothers. Therefore, we conducted the present cross-sectional survey with larger sample and interviewed both children and their mothers.

No report has examined the relationship between phthalate exposure and children’s IQ. We hypothesized that phthalate metabolites in urine might be related to children’s IQ independent of maternal IQ.

## Materials and Methods

### Study protocol and recruitment

This study was conducted between April and October of 2008. In a second-year study named The Effects of Pollution on Neurobehavioral Development and Future Policies to Protect Our Children, funded by the Eco-Technopia 21 project, we assessed lead, mercury, and manganese in blood as well as cotinine and phthalate metabolites in urine (Cho et al. 2010). We assessed the children’s neurocognitive functioning, including general intelligence, attention, and concentration. We interviewed the children’s mothers to evaluate maternal IQ and child developmental state as well as to screen for the presence of several psychiatric disorders. Questionnaires were also given to parents asking for information about environmental surroundings, such as the type of housing, number of rooms in the house, pesticide and organophosphate exposure, distance from the road, proximity to industry, presence of incineration plants in the neighborhood, quality of air, pets, exposure to indirect smoking, and diet.

The Institutional Review Board of Seoul National University Hospital approved the study protocol. Participants were recruited from Seoul (metropolitan area, 605.3 km^2^; population, 11,000,000) Seongnam (suburban area, 141.8 km^2^; population, 971,150), Incheon (industrial area, 1002.07 km^2^; population, 2,758,431), Ulsan (industrial area, 1,057 km^2^; population, 1,129,827), and Yeoncheon (rural area, 695.3 km^2^, population, 44,974). From each area, we chose one to three schools (total of nine) that best represented the local environment. An invitation to participate in the study was sent to third- and fourth-grade children and their parents. After being given a detailed explanation, 667 children/parents of 1,000 gave informed consent to participate in the study (participation rate, 66.7%). Among fourth- through sixth-grade elementary school students in participating schools, the sex ratio and the mean age of the children were not different between participants and nonparticipants.

### Measuring children’s cognitive functioning

The children were individually administered the abbreviated form of the Korean Educational Development Institute–Wechsler Intelligence Scale for Children (KEDI-WISC) (Park et al. 1996), which consists of the following scales: vocabulary, arithmetic, picture arrangement, and block design tests. This form of the KEDI-WISC has been validated with Korean children and is used to determe global estimates of intellectual functioning. Kim and Kim (1986) have reported that the correlations between this abbreviated form and the original one ranged from 0.89 to 0.92 on the four sub-scales; the correlations between the two forms for the verbal, performance, and Full Scale IQs (FSIQs) were 0.97, 0.96, and 0.98, respectively. Scores obtained on an abbreviated battery are highly correlated with the WISC FSIQ scores (Kaufman 1976). The KEDI-WISC is useful in determining children’s IQ, as discussed previously (Kim et al. 2009b). The sums of the age-adjusted *t*-scores for arithmetic and vocabulary were used to estimate Verbal IQ (VIQ), and the sums of scores for block design and picture arrangement were used to estimate Performance IQ (PIQ).

For the testing, 23 examiners were trained for 8 hr by a licensed clinical psychologist with 20 years of experience in the field (M.-S.S.). Additionally, examiners had a 2-hr meeting before the beginning of the session to verify testing skills. Research nurses supervised the tests in every school at each site. All of the raw data were reviewed by the research nurse and a child psychiatrist (H.-W.K.) to confirm the IQ scores of each subject.

### Measuring maternal cognitive function

Examiners, unaware of the children’s IQ levels, administered the short form of the Korean Wechsler Adult Intelligence Scale (K-WAIS) (Lim et al. 2000) to the children’s mothers. This test consists of vocabulary, arithmetic, picture arrangement, and block design tests. Scores obtained on abbreviated batteries have been found to be highly correlated with FSIQ scores (Silverstein 1990). The sum of the age-adjusted *t*-scores for arithmetic and vocabulary was used to estimate VIQ, and the scores for block design and picture arrangement were used to estimate PIQ.

### Determining phthalate metabolites in urine

The metabolites measured in this study included one primary metabolite of dibutyl phthalate, mono-*n*-butyl phthalate (MBP), and two secondary metabolites of DEHP, mono-2-ethylhexyl phthalate (MEHP) and mono(2-ethyl-5-oxohexyl)phthalate (MEOHP).

We collected urine using a paper cup from each child between 0900 and 1100 hours at the school. Immediately after collection, we placed the urine in containers (high-clarity polypropylene Falcon tubes) and stored them at −20°C until the samples were analyzed. Field blanks consisted of purified water. Later, the samples were brought to room temperature and vortexed after thawing. Next, 500 μL urine was buffered with 30 μL 2.0 M sodium acetate (pH 5.0) and spiked with a mixture of isotope phthalate monoester standards (100 ng/mL) and 10 μL β-glucuronidase. The sample was incubated at 37°C for 3 hr to deconjugate the glucuronidated phthalate metabolites. After incubation, 100 μL 2N HCl was added to collect the phthalate monoester. The extract was dried with nitrogen gas and reconstituted with 1 mL HPLC-grade H_2_O in a 2-mL glass vial. One blank and one quality-control (QC) sample were included in each batch of samples. The QC sample was spiked with pooled urine and a mixture of phthalate monoester standard (100 ng/mL). The supernatants were purified using solid-phase extraction with disposable Agilent SB C18 1.8 μm (2.1 × 50 mm) columns (Agilent, Santa Clara, CA, USA). The mobile phase was 0.1% acetic acid water:0.01% acetic acid acetonitrile (90:10, vol/vol) at a flow rate of 0.25 mL/min, and the eluates were monitored at target masses of 221, 293, and 291, and internal standard (ISTD) masses of 225, 297, and 295. The monoester phthalates were measured using high-performance liquid chromatography tandem mass spectrometry (Agilent 6410 triple Quad LCMS; Agilent) (Kho et al. 2008). The method detection limit of phthalate metabolites was 0.016, 0.045, and 0.440 μg/L for MEHP, MEOHP, and MBP, respectively. We added each value (micrograms per liter) for summing two secondary metabolites of DEHP, MEHP and MEOHP. We used a calibration curve (*R*^2^ > 0.995) for QC in every batch following the QC protocol of the mass spectrometry.

For creatinine measurement, CREA (Roche, Indianapolis, IN, USA) reagent was used with a Hitachi 7600 machine (Hitachi, Tokyo, Japan) with a kinetic colormetric assay (rate-blanked and compensated). We used value (micrograms per liter)/creatinine (grams per liter) for dilution correction in the analyses.

### Statistical analyses

We used Student *t*-tests or analyses of variance (ANOVAs) for testing continuous variables, and the chi-square test for categorical variables. The associations between urine phthalate (MEOHP, MEHP, and MBP) concentration and WISC IQ scores were assessed using linear regression analysis. Because the distribution of the metabolite was skewed in the sample, we used log-transformed values (ln) in the analyses.

Using the significant variables from the above analysis, we built multiple regression models. In the regression analyses, the IQ score was the primary dependent variable, and concurrently measured urine phthalate concentrations were the primary independent variables. Regression analyses were performed using a set of covariates based on established predictors of children’s cognitive functioning. Models were adjusted for developmental, socioeconomic, and familial influences on IQ variables such as age, sex, birth weight, history of breast-feeding, residential area, socioeconomic status (SES), and paternal educational level. Because maternal IQ might serve as an indicator of the quality of the home environment and stimulation in the home, we present models with and without the maternal IQ.

Because endocrine disruptors are known to affect the sexes differently, we divided the participants into two groups by sex and explored the relationship between IQ score and urine metabolites (quartile) using ANOVA.

Sex, SES (yearly income separated into above and below average), history of breast-feeding (yes or no), paternal education (above and below high school), indirect smoking (yes or no), smoking during pregnancy (yes or no), and the five residential areas were categorical variables. All analyses were considered to be statistically significant for *p*-values < 0.05. Statistical analyses were performed using SPSS 17.0 for Windows (SPSS, Chicago, IL, USA).

## Results

### Participant characteristics

The geographic distribution of the study participants was as follows: Seoul, *n* = 279 (41.8%); Seongnam, *n* = 73 (10.9%); Incheon, *n* = 126 (18.9%); Ulsan, *n* = 113 (16.9%); and Yeoncheon, *n* = 76 (11.4%). The number of children recruited from each city differed significantly [χ^2^ = 214.5; degrees of freedom (df) = 4; *p* < 0.001]. Of the 667 subjects, sufficient urine amounts to measure phthalates were available for 658 (98.7%). We excluded an additional 34 participants from the analyses because of low birth weight (< 2.5 kg, *n* = 31), a history of neonatal hypoxia (*n* = 1), and the presence of a seizure disorder (*n* = 2).

The mean (± SD) age of the participants was 9.05 ± 0.72 years (range, 8–11), and 48.4% were female. Table 1 summarizes the demographic characteristics of the subjects. We found significant differences in the background characteristics between the children included (*n* = 621) and excluded (*n* = 46) from the analyses with respect to age, gestational age at birth, and birth weight. All other variables were comparable between included and excluded children.

The mean (± SD) full-scale KEDI-WISC for the total sample was 110.7 ± 15.0 (range, 49–152), the mean VIQ was 23.2 ± 5.5 (range, 6–36), and the mean PIQ was 23.5 ± 5.3 (range, 3–37). We found no significant interobserver variability in the children’s mean IQ scores. The girls performed worse than the boys on the FSIQ [mean difference = 2.8; 95% confidence interval (CI), 0.4–5.1; *p* = 0.02] and VIQ measures (mean difference = 1.3; 95% CI, 0.4–2.1; *p* = 0.004).

We obtained significant correlations between children’s IQ and both birth weight and maternal IQ (respectively, *r* = 0.12, *p* = 0.005; and *r* = 0.39, *p* < 0.001). We found significant relations between child FSIQ and educational level of the father (more than high school, 112.1 ± 14.8; less than high school, 102.7 ± 15.4; *p* = 0.004), sex (boys, 112.0 ± 14.7; girls, 109.3 ± 15.2; *p* = 0.02), SES (above average, 113.0 ± 15.0; below average, 108.7 ± 14.6; *p* = 0.01), and history of breast-feeding (breast-feeding, 113.0 ± 14.1; not breast-feeding, 108.8 ± 16.1; *p* = 0.01) by *t*-test. FSIQ was different from the residential area (Seoul, 114.6 ± 13.5; Sungnam, 106.1 ± 12.3; Incheon, 106.1 ± 14.7; Ulsan, 112.1 ± 16.1; Yeonchun, 106.0 ± 16.8; *p* < 0.001) by ANOVA. Thus, we used these as covariates in multivariate analysis.

All of the urinary phthalate biomarkers exceeded the limits of detection (Table 2). The geometric mean (ln) concentrations of MEHP, MEOHP, and MBP were 21.3 μg/L [geometric SD (GSD) = 2.2; range, 0.5–445.4], 18.0 μg/L (GSD = 2.4; range, 0.07–291.1), and 48.9 μg/L (GSD = 2.2; range, 2.1–1645.5), respectively. The mean (± SD) concentration of urine creatinine was 1.14 ± 0.51; range, 0.05–2.83) g/L.

### The relationship between phthalate metabolites and IQ

We used linear regression analysis to determine the relationship between phthalate metabolites and IQ score. Additionally, we attempted to control for the possible effects of dilution (Engel et al. 2009). First, we used creatinine-corrected concentrations. Next, we analyzed the data twice including or excluding the observations with urinary creatinine values > 0.2 g/L to eliminate data from extremely dilute urine samples (*n* = 11). Because the results of these two analyses did not differ, we have reported the results from all observations (*n* = 621). Because urine phthalates were not distributed normally, we used natural log-transformed values in analyses.

We first analyzed the influence on each measure of children’s IQ to choose which covariates to include in our multiple regression models (Table 3). We calculated the sum of MEHP and MEOHP because both are metabolites of DEHP. MEHP, MEOHP, and the sum of DEHP metabolites were significantly related to FSIQ, VIQ, and the vocabulary and block design scores; MBP was significantly related to the vocabulary and block design scores.

For FSIQ, VIQ, and the vocabulary and block design scores, we used multiple linear regression analysis with covariates to control for confounding effects (Table 4). We used log-*e* values in analyses. Models are adjusted for developmental, socioeconomic, and familial influences on IQ variables. We present the two models, without maternal IQ (model 1) or with maternal IQ (model 2) as a covariate. In model 1, the results were similar to those from univariate analyses, except for an insignificant relationship between block design and MBP. Finally, we added maternal IQ as a covariate. The relations between MEHP, MEOHP, and the sum of DEHP metabolites to the vocabulary subscore were still significant (*p* < 0.001). Therefore, a one-level change in the ln MEHP, MEOHP, or sum of DEHP metabolites altered the vocabulary subscore by −0.501, −0.444, and −0.529, respectively, independently of all of the covariates, including maternal IQ.

**Figure 1:**
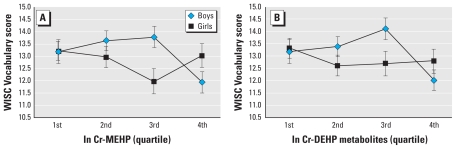
Adjusted mean WISC Vocabulary score estimated by MEHP (*A*) and DEHP metabolites (*B*) quartiles of ln creatinine (Cr)-corrected urine concentrations. Adjusted means differed significantly between the second and fourth quartiles and between the third and fourth quartiles of ln creatinine-corrected MEHP and ln creatinine-corrected DEHP metabolites for boys (*p* < 0.05). Among girls, there were no significant associations between these variables. Estimated means are adjusted for age, sex, birth weight, history of breast-feeding, residential area, paternal education, SES, and maternal IQ. Error bars are SEs.

Because endocrine disruptors are known to affect the sexes differently, we plotted the vocabulary scores by sex (Figure 1). In boys, vocabulary scores were 1.69 score lower (95% CI, 0.5–2.9) and 1.85 score lower (95% CI, 0.66–3.0) in the fourth quartile of ln creatinine-adjusted MEHP (highest) compared with the second and third quartiles (adjusted means: fourth quartile, 11.9; 95% CI, 11.1–12.8; second quartile, 13.6; 95% CI, 12.8–14.4; third quartile, 13.8; 95% CI, 12.9–14.6) by ANOVA (*p* = 0.01). Also, in boys, vocabulary scores were 1.36 score lower (95% CI, 0.20–2.51) and 2.08 score lower (95% CI, 0.90–3.26) in the fourth quartile of ln creatinine-adjusted DEHP metabolites (highest) compared with the second and third quartiles (adjusted means: fourth quartile, 12.0; 95% CI, 11.2–12.9; second quartile, 13.4; 95% CI, 12.6–14.2; third quartile, 14.1; 95% CI, 13.2–15.0) by ANOVA (*p* = 0.006). However, among girls, we found no significant association between these variables.

## Discussion

In this study we investigated the relationship between environmental exposure to phthalates and children’s IQ scores. To the best of our knowledge, this is the first study to show a negative association between IQ and urine phthalates in humans. A child’s IQ can be influenced by genetic, familial, educational, and social factors (Bellinger 2000, 2004, 2008). These factors might confound any observed association between the exposure of interest and the outcome (Mink et al. 2004; Sattler 2001). Therefore, we tried to determine whether phthalates were inversely associated with children’s cognition independent of potential confounders. Before we adjusted for maternal IQ, we found inverse relationships between MEHP, MEOHP, and the sum of secondary metabolites for DEHP and the children’s FSIQ, VIQ, and vocabulary and block design scores. MBP was also negatively associated with vocabulary score. Maternal IQ is a well-known confounder of children’s IQ (Mink et al. 2004; Sattler 2001); however, adjusting for a confounder that has a stronger effect than the variable of interest can lead to underestimations in the true effect of this variable (in this case, metabolite levels) (Bellinger 2008).

In this study, controlling for maternal IQ had a large impact on the effect size of the association between phthalates and IQ. It might work as a confounder if maternal IQ and the level of phthalates in children are related to each other. Maternal IQ might have influenced the children’s environmental exposure to these substances, which would be reflected by the level of phthalate metabolites. At the same time, maternal IQ affects child IQ and therefore affects the relationship between child IQ and phthalate level, although after adjusting for maternal IQ, MEHP, MEOHP, and the sum of secondary metabolites of DEHP showed inverse relationships with children’s vocabulary scores in this study. However, because there could be a residual confounding of maternal IQ, we cannot say that this study provides evidence of a definitive relationship of phthalates with children’s IQ independent of maternal IQ.

Children are of special concern in terms of phthalate exposure because of their neurodevelopmental state. Exposure to neurotoxic agents is more detrimental to children because of critical periods of vulnerability for the developing nervous system (Rice and Barone 2000). Neurotoxic agents could disrupt the temporal and regional emergence of critical developmental processes even after birth (i.e., synapse formation and myelination).

There are some limitations of this study. First, although at least 16 phthalate metabolites in urine can be measured (Kato et al. 2005), we measured only three, based on children’s likely exposure, availability of standard samples, and technical experience of the analyses. Second, using a single measurement might not have the same implications as examining the level and severity of chronic exposure. Phthalates are rapidly metabolized and excreted, and a single spot-urine measurement may not reflect long-term exposure; however, several studies have reported low to moderate consistency of phthalate measurements over time (Fromme et al. 2007; Hauser et al. 2004; Hoppin et al. 2002). When sources or exposure patterns are consistent, we can assume that a single measurement reflects a typical measurement. Even so, we have to be careful when expanding our results to other phthalates because the vulnerability of the developing brain to chemicals reportedly depends on the duration of exposure (Tanida et al. 2009). Our cross-sectional design cannot rule out the possibility of misclassification between exposure and outcome, so further research using prospective designs is needed; however, even in a prospective study, maternal IQ could still contribute to a child’s exposure opportunities such that an observed association would not be unequivocally causal.

Third, we did not evaluate maternal levels of exposure. To our knowledge, there is no report presenting phthalate exposure data from both children and their mothers. If we were to measure the exposure of the family simultaneously, then we could clarify the effect of the coexposure from other confounding factors as well. Fourth, because maternal IQ had a large effect size on the associations, the quality of the home environment and stimulation provided in the home could be potential confounders. One of the limitations of our study is that we did not gather systemic measures (e.g., the Caldwell Home Observation for Measurement of the Environment inventory) (Zevalkink et al. 2008) of these additional potential confounders.

The mechanism that might cause negative effects of phthalates on neural development is uncertain (Engel et al. 2009). MEHP activates peroxisome proliferator-activated receptor-α (PPARα) via the activation of nuclear receptors (Roberts et al. 1997). Ligands of PPAR play roles in lipid metabolism, cellular proliferation, and the inflammatory response and are widely expressed in inflammatory, dendritic, and endothelial cells (Kota et al. 2005). Its signal transduction pathway has recently been implicated in the progression of neuro-degenerative and psychiatric diseases and its relation to cognitive function (van Neerven et al. 2008). The activation of PPARα by phthalate monoesters induces rodent hepato-carcinogenesis (Hays et al. 2005; Peters et al. 1997). PPARs have been observed in developing neural tubes (Braissant and Wahli 1998) and may alter lipid metabolism in the brain (Xu et al. 2007). Furthermore, phthalates are suspected of interfering with the thyroid hormone system (Ghisari and Bonefeld-Jorgensen 2009). Although human data are lacking, *in vitro* and *in vivo* studies suggest that phthalate exposure is associated with altered thyroid functioning (Breous et al. 2005; Hinton et al. 1986; Pereira et al. 2007; Wenzel et al. 2005). Phthalate exposure may be associated with altered thyroid activity in adult men (Meeker et al. 2007) and in pregnant women (Huang et al. 2007). Just as neonatal hypothyroidism affects cognition, subclinical hypothyroidism can affect children’s cognition (Baldini et al. 1997; Monzani et al. 1993; Zhu et al. 2006).

Cognitive functioning is related to the regulation of the neurotransmitter system. Genetic variation in the catechol-*O*-methyltransferase (*COMT*) gene at several loci affects normal cognitive functioning in children (Barnett et al. 2009). Several animal studies have revealed that the dopamine system in the central nervous system is affected by phthalates. Low-dose phthalates can cause the loss of midbrain dopaminergic neurons, decrease tyrosine hydroxylase–biosynthetic activity (Tanida et al. 2009), and impair tyrosine hydroxylase immunoreactivity (Ishido et al. 2004).

We assumed that potential effects of phthalates on cognitive functioning would differ by sex because phthalates are well-known endocrine disruptors with estrogen-like effects (Kamrin 2009; Shea 2003). Relatively low doses of DEHP resulted in different dose–response curves by sex in an animal study (Andrade et al. 2006). Boys and girls may demonstrate opposite patterns of association between phthalate level and performance with improved motor performance with increasing concentrations of phthalate metabolites among boys in human subjects (Engel et al. 2009). We found a different pattern for the association of phthalates with vocabulary scores by sex, which concurs with previous studies of different results according to sex (Hatch et al. 2008; Wolff et al. 2008).

Children can be exposed to phthalates via ingestion, inhalation, and absorption through normal skin (Janjua et al. 2007) as well as via exposure to medical devices contaminated with phthalates (Breous et al. 2005). In our study, after controlling for maternal IQ and other covariates, the results show an inverse relationship between phthalate metabolites and IQ scores; however, given the limitations inherent in cross-sectional epidemiology, prospective studies are needed to fully explore these associations.

## Figures and Tables

**Table 1:** Demographic characteristics of the subjects.

Characteristic	All participants (*n*= 667)	Site					Children selected for analysis (*n*= 621)	Children excluded^a^ from the analysis (*n*= 46)	*p*-Value
		Seoul (*n*= 262)	Sungnam (*n*= 66)	Incheon (*n*= 119)	Ulsan (*n*= 104)	Yeonchun (*n*= 70)			
Age [years (mean ± SD)]	9.0 ± 0.7	9.0 ± 0.7	8.9 ± 0.6	9.0 ± 0.7	9.0 ± 0.7	9.5 ± 0.7	9.0 ± 0.7	9.3 ± 0.7	0.038
Female sex [*n* (%)]	323 (48.4)	144 (51.6)	36 (49.3)	59 (46.8)	49 (43.4)	35 (46.1)	302 (48.6)	21 (45.7)	0.761
Indirect smoking exposure [*n* (%)]	355 (53.2)	134 (53.4)	43 (68.3)	82 (66.7)	58 (53.7)	38 (61.3)	330 (53.1)	25 (54.3)	0.874
Yearly income > $25,000 [*n* (%)]	374 (56.1)	175 (69.7)	27 (61.4)	62 (50.0)	77 (70.7)	33 (51.6)	344 (55.4)	30 (65.2)	0.519
Maternal education									0.973
< High school [*n* (%)]	26 (3.9)	9 (3.6)	3 (6.5)	6 (5.0)	4 (3.7)	4 (6.6)	24 (3.9)	2 (4.3)	
≥ High school [*n* (%)]	559 (83.8)	241 (96.4)	43 (93.5)	113 (95.0)	105 (96.3)	57 (93.4)	517 (83.3)	42 (91.3)	
Paternal education									0.576
< High school [*n* (%)]	23 (3.4)	7 (2.8)	0 (0)	6 (5.1)	4 (3.60)	6 (9.8)	22 (3.5)	1 (2.2)	
≥ High school [*n* (%)]	564 (84.6)	246 (97.2)	43 (100)	112 (94.9)	108 (96.4)	55 (90.2)	522 (84.1)	42 (91.3)	
Maternal age at pregnancy (mean ± SD)	28.57 ± 3.9	28.8 ± 3.2	28.3 ± 4.3	28.1 ± 4.2	28.6 ± 4.0	28.7 ± 4.9	28.56 ± 3.9	28.66 ± 3.3	0.868
Smoking during pregnancy [*n* (%)]	4 (0.6)	1 (0.4)	1 (2.0)	1 (0.8)	0	1 (1.6)	4 (0.6)	0	0.572
Alcohol during pregnancy [*n* (%)]	24 (3.6)	9 (3.5)	4 (7.8)	7 (5.6)	2 (1.9)	2 (3.2)	22 (3.5)	2 (4.3)	0.852
Gestational age at delivery [weeks (mean ± SD)]	39.7 ± 1.4	39.7 ± 1.3	39.5 ± 1.7	39.7 ± 1.3	39.7 ± 1.3	39.6 ± 1.5	39.9 ± 0.8	37.30 ± 3.3	< 0.001
Birth weight [kg (mean ± SD)]	3.2 ± 0.5	3.3 ± 0.5	3.1 ± 0.5	3.2 ± 0.4	3.2 ± 0.5	3.2 ± 0.5	3.3 ± 0.4	2.4 ± 0.6	< 0.001
Urinary phthalate [GM (GSD)]									
MEHP		17.3 (2.5)	27.6 (2.0)	24.6 (2.0)	29.9 (1.7)	17.2 (2.0)			
MEOHP		15.1 (2.7)	24.6 (2.1)	18.9 (2.3)	26.9 (1.7)	13.1 (2.1)			
MBP		46.3 (2.2)	57.8 (1.8)	42.3 (2.4)	77.4 (1.6)	32.9 (2.2)			

Geometric mean (GM) indicates log *e*.

a Children with low birth weight (< 2.5 kg, *n*= 31), seizure disorders (*n*= 2), hypoxia (*n*= 1), and incomplete data (*n* = 12, no urine sample or no IQ score) were excluded from the main analyses.

**Table 2:** Urinary phthalate biomarkers among the participants.

Phthalate	Percent > LOD	Geometric mean (GSD)	Minimum	Percentile			Maximum
				25th	50th	75th	
MEHP	100.0	21.3 (2.3)	0.506	11.832	24.721	46.138	445.376
MEOHP	100.0	18.0 (2.4)	0.065	9.841	20.556	39.506	291.111
MBP	100.0	48.9 (2.2)	2.076	24.869	50.470	93.487	1645.498

LOD, limit of detection. Geometric mean indicates log *e*.

**Table 3:** Univariate analysis of log-*e* creatinine-adjusted urinary concentrations of phthalate biomarkers with IQ tests.

	β-Value for log biomarkers (SE)						
Phthalate	FSIQ	VIQ	PIQ	Math	Vocabulary	Block design	Picture
MEHP	−1.93 (0.74)**	−0.91 (0.27)**	−0.13 (0.26)	−0.10 (0.14)	−0.80 (0.17)#	−0.37 (0.16)*	0.17 (0.15)
MEOHP	−1.74 (0.68)*	−0.81 (0.25)**	−0.17 (0.24)	−0.11 (0.13)	−0.68 (0.16)#	−0.31 (0.15)*	0.12 (0.14)
MEHP + MEOHP	−1.96 (0.74)**	−0.93 (0.27)#	−0.16 (0.26)	−0.01 (0.14)	−0.80 (0.17)#	−0.37 (0.16)*	0.17 (0.15)
MBP	−0.38 (0.76)	−0.46 (0.28)	0.40 (0.27)	0.03 (0.15)	−0.47 (0.18)*	0.02 (0.17)*	0.25 (0.16)

Picture refers to picture arrangement.

* *p* < 0.05,

** *p* < 0.01,

# *p* < 0.001.

**Table 4:** Multiple regression analysis of the relationship of phthalates with IQ adjusted for influences of covariates.

	β-Values for log biomarkers											
	FSIQ			VIQ			Vocabulary			Block		
Model/phthalate	β-Value (SE)	95% CI	*p*-Value	β-Value (SE)	95% CI	*p*-Value	β-Value (SE)	95% CI	*p*-Value	β-Value (SE)	95% CI	*p*-Value
Model 1												
MEHP	−2.0 (0.8)	−3.5 to −0.4	0.016	−0.8 (0.3)	−1.4 to −0.2	0.008	−0.8 (0.2)	−1.2 to −0.5	< 0.001	−0.4 (0.2)	−0.7 to −0.1	0.048
MEOHP	−2.2 (0.7)	−3.6 to −0.8	0.003	−0.8 (0.3)	−1.3 to −0.3	0.003	−0.7 (0.2)	−1.1 to −0.4	< 0.001	−0.4 (0.2)	−0.7 to −0.1	0.019
MEHP + MEOHP	−2.3 (0.8)	−3.8 to −0.7	0.005	−0.9 (0.3)	−1.5 to −0.3	0.003	−0.9 (0.2)	−1.2 to −0.5	< 0.001	−0.4 (0.2)	−0.8 to −0.1	0.023
MBP	−0.6 (0.8)	−2.2 to 1.0	0.440	−0.5 (0.3)	−1.1 to 0.1	0.136	−0.6 (0.2)	−0.9 to −0.2	0.005	−0.1 (0.2)	−0.5 to 0.3	0.625
Model 2												
MEHP	−0.2 (0.8)	−1.8 to 1.4	0.795	−0.3 (0.3)	−0.9 to 0.2	0.283	−0.5 (0.2)	−0.8 to −0.2	0.01	0.1 (0.2)	−0.3 to 0.4	0.748
MEOHP	−0.5 (0.8)	−2.0 to 1.2	0.552	−0.3 (0.3)	−0.9 to 2.4	0.264	−0.4 (0.2)	−0.8 to −0.1	0.015	−0.1 (0.2)	−0.4 to 0.3	0.799
MEHP + MEOHP	−0.4 (0.8)	−2.0 to 1.2	0.629	−0.4 (0.3)	−1.0 to 0.2	0.220	−0.5 (0.2)	−0.9 to −0.1	0.007	−0.1 (0.2)	−0.4 to 0.4	0.997
MBP	0.4 (0.1)	−1.4 to 2.1	0.683	−0.1 (0.3)	−0.8 to 0.6	0.769	−0.3 (0.1)	−0.7 to 0.2	0.191	0.1 (0.2)	−0.4 to 0.4	0.937

Model 1 was adjusted for age, sex, birth weight, history of breast-feeding, residential area, paternal education, SES. Model 2 was adjusted as for model 1 plus maternal IQ (using the same subset).
